# Spatio-temporal patterns of stream methane and carbon dioxide emissions in a hemiboreal catchment in Southwest Sweden

**DOI:** 10.1038/srep39729

**Published:** 2017-01-03

**Authors:** Sivakiruthika Natchimuthu, Marcus B. Wallin, Leif Klemedtsson, David Bastviken

**Affiliations:** 1 Department of Thematic Studies–Environmental Change, Linköping University, 581 83 Linköping, Sweden; 2 Department of Earth Sciences, Uppsala University, 752 36 Uppsala, Sweden; 3 Department of Ecology and Genetics/Limnology, Uppsala University, 752 36 Uppsala, Sweden; 4 Department of Earth Sciences, University of Gothenburg, 405 30 Gothenburg, Sweden

## Abstract

Global stream and river greenhouse gas emissions seem to be as large as the oceanic C uptake. However, stream and river emissions are uncertain until both spatial and temporal variability have been quantified. Here we investigated in detail the stream CH_4_ and CO_2_ emissions within a hemiboreal catchment in Southwest Sweden primarily covered by coniferous forest. Gas transfer velocities (*k*_*600*_), CH_4_ and CO_2_ concentrations were measured with multiple methods. Our data supported modelling approaches accounting for various stream slopes, water velocities and discharge. The results revealed large but partially predictable spatio-temporal variabilities in *k*_*600*_, dissolved gas concentrations, and emissions. The variability in CO_2_ emission was best explained by the variability in *k,* while dissolved CH_4_ concentrations explained most of the variability in CH_4_ emission, having implications for future measurements. There were disproportionately large emissions from high slope stream reaches including waterfalls, and from high discharge events. In the catchment, stream reaches with low slope and time periods of moderate discharge dominated (90% of area and 69% of time). Measurements in these stream areas and time periods only accounted for <36% of the total estimated emissions. Hence, not accounting for local or episodic high emissions can lead to substantially underestimated emissions.

Inland waters emit significant amounts of greenhouse gases such as methane (CH_4_) and carbon dioxide (CO_2_) to the atmosphere^1^,^2^,^3^,^4^,^5^,^6^. Despite their low areal coverage, streams are one of the most important contributors to the total aquatic CO_2_ emissions and the estimated global CO_2_ emissions from streams and rivers (1.8 Pg C yr^−1^) exceed the corresponding emissions from lakes and reservoirs (0.3 Pg C yr^−1^)^2^. Moreover, global stream CO_2_ emissions are in the same order of magnitude as the estimated land C sink of 2.6 Pg C yr^−1^ or oceanic C uptake of 2.3 Pg C yr^−1 ^7. The disproportionately large contribution from streams is partly a function of higher concentrations of CO_2_ observed in streams than in lakes^8^,^9^. The gas transfer velocities (*k*) observed in streams are also usually higher than in lakes due to the turbulence generated when water is moving along a stream channel. Consequently, some of the highest reported *k* values have been observed in small streams^3^. Even though stream emissions appear to be large contributors to landscape aquatic CO_2_ budgets with high per m^2^ emissions^10^, they are often neglected in landscape level carbon budgets due to the lack of data. Furthermore, stream CH_4_ emissions are poorly understood although there is a growing awareness of their importance^11^,^12^. Global stream emissions have been estimated to be 27 Tg CH_4_ yr^−1^ by Stanley *et al*.^6^ and it has been suggested that CH_4_ emissions from streams could be even more variable in space and time than CO_2_ emissions^6^,^13^.

Previous stream emission estimates often rely on data with poor resolution in space and time. Yet, stream emissions are known to be very variable. The spatial variability in gas emissions has been linked to highly variable *k* which is a result of e.g. stream channel morphology, channel slope, water velocity etc.^14^,^15^,^16^. In addition, dissolved gas concentrations along a stream network are also often highly variable as a result of both gas input (influenced by surrounding land-use including upstream wetlands, soil characteristics, groundwater inputs, etc.^5^,^11^,^17^,^18^,^19^,^20^,^21^,^22^), and gas loss (dependent on *k* which regulates the vertical gas exchange^13^). Furthermore, temporal variations in gas concentrations and emissions have been linked to variations in stream discharge^23^,^24^. A variable discharge causes changes in the ground water level and hence the input of gases to the stream^17^,^25^. Discharge events like snow melt and rainstorm events can have a rapid effect on the stream gas concentrations and emissions either due to dilution or increased supply of dissolved gases^12^,^25^,^26^,^27^. Variability in discharge could also affect the *k* through changes in turbulence which affect the emissions^15^. Diel cycles in CO_2_ concentrations as a result of photosynthesis and/or mineralisation processes have also been recorded in streams^18^,^28^. Thus, large spatio-temporal variability in stream gas emissions could be expected while the present data rarely allow analysis of how this variability influences yearly stream network emissions. Therefore, there is a need for long-term studies of whole catchment stream network greenhouse gas emissions including both spatial and temporal dimensions.

Another dilemma is that most large scale aquatic CO_2_ emission estimates are based on indirect methods where CO_2_ is estimated using temperature, pH and total alkalinity^2^,^29^. Such indirect methods have now been shown to overestimate CO_2_ concentrations, in organic rich, acidic, or low alkalinity waters^30^. There have been attempts to reduce this bias by not considering data from water with a pH below 5.4^2^,^29^. This resolves some dilemmas but also means that acid waters were not accounted for. In any case, indirect estimates of concentrations lead to great uncertainties and more data from direct, continuous measurements are needed. Accordingly, a growing number of recent large scale studies also focus on direct methods to measure concentrations^5^,^19^,^20^,^21^. In this study we combined direct measurements of *k* (using a tracer gas approach) with direct determination of CH_4_ and CO_2_ concentrations during a two-year study period in the stream network of a hemiboreal catchment in Southwest Sweden (Fig. 1). The overall aim was to assess and account for spatio-temporal dynamics when estimating stream network CH_4_ and CO_2_ emissions. To do this we (1) measured the spatio-temporal variability in *k* in key reaches of the stream network (2) used the measurements to develop a locally validated model for *k* for the whole stream network (3) derived stream network CH_4_ and CO_2_ emissions from directly measured concentrations and the modelled *k,* accounting for the identified spatio-temporal variability and (4) quantified the importance of turbulent sections and high discharge periods.

## Results

### Measured and modelled gas transfer velocities

**Figure 1:**
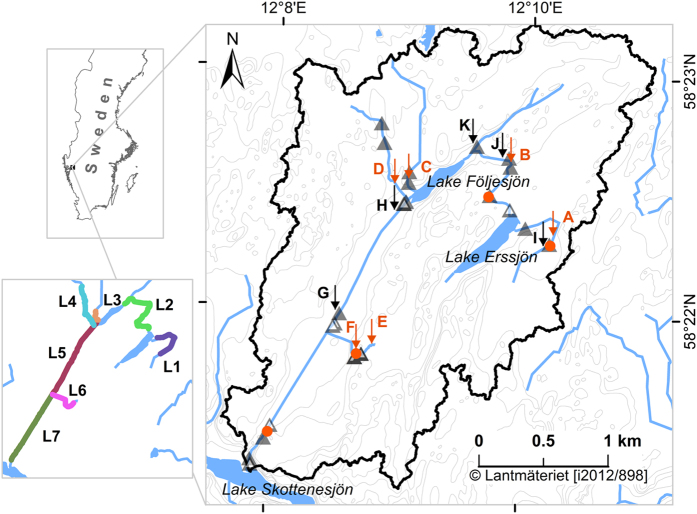
A map of the Skogaryd Research Catchment (SRC) showing the studied streams, the catchment boundaries and sampling locations. Red arrows denote the reaches (**A–F**) where propane injections were made and the triangles denote the locations of CO_2_ sensor chambers (open triangles–2012, closed triangles–2013). Black arrows (G–K) denote points where CO_2_ emissions were directly measured for validation using the drifting/floating chamber method (see Supplementary Information). The locations of the four discharge monitoring stations are given as red circles. The inset map shows the different location categories (L1–L7) made for the purpose of describing spatial variability. The figure was created with ArcMap 10.3.1 available from http://www.esri.com/. The background map was obtained from Lantmäteriet (National Land Survey of Sweden) and published under the copyright agreement i2012/898 with Linköping University.

Gas transfer velocities (*k*) depend on the diffusivity of the gas and the temperature influencing the viscosity of the water. Hence, *k* values are related to Schmidt numbers representing the ratio of the kinematic viscosity of water to the diffusion coefficient of the gas in focus. For comparison across temperatures and gases, *k*_*600*_, representing *k* normalised to a Schmidt number of 600 (corresponding to the Schmidt number for CO_2_ at 20 °C^31^), is often used. The *k*_*600*_ measured in six stream reaches using propane injections ranged from 0.2 to 558.7 m d^−1^, respectively (Table 1). Reaches characterised by steep sections and waterfalls (B, C, and F in Fig. 1) had much higher *k*_*600*_ than reaches having almost straight channels with flat topography (A, D and E in Fig. 1, see Table 1 for mean and ranges). Additional description of the results from the propane injections is given in the Supplementary Information.

**Figure 2:**
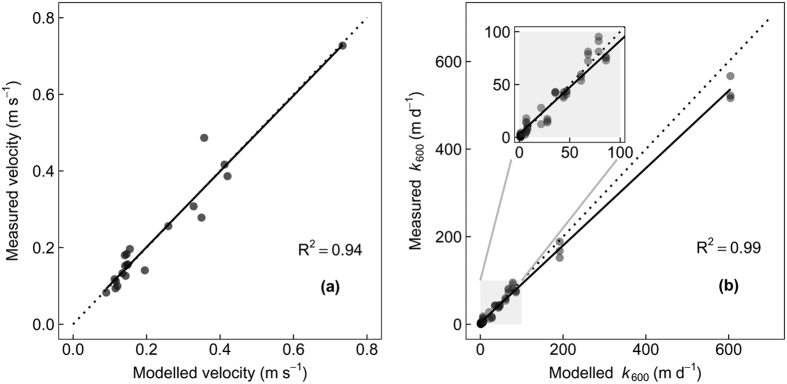
Scatter plots of modelled velocity (**a**) and *k*_*600*_ (**b**) against the corresponding measured values. All *p* values were < 0.001. The insets in panel (**b**) shows the fit in the lower range of *k*_*600*_. The modelled *k*_*600*_ was multiplied by a factor of 0.89 to avoid overestimates in higher ranges of modelled values (see text). Darker symbol colour indicates data point overlaps.

Bivariate relationships between *k*_600_ and discharge or water velocity were strong for reaches with higher slopes but were weak or absent in the flatter reaches (Fig. S1; see details on velocity estimation in the Supplementary Information). However, multiple linear regression models of *k*_*600*_ as a function of water velocity and slope generated strong relationships with adjusted R^2^ of 0.92 (*p* < 0.001; Table 2), and a close correspondence between predictions and observations for *k*_*600*_ (Fig. 2). However, the model slightly overestimated *k*_*600*_ in the higher range. To correct this, we multiplied the modelled *k*_*600*_ by a factor of 0.89 obtained from the slope of the linear regression between measured and modelled values (Fig. 2b). This corrected model was used to estimate daily *k*_*600*_for all reaches of the stream network. The division of the stream network into 84 reaches, each covering a change in elevation of 0.5 m, is described in the Supplementary Information. The reaches were longer in flat areas and shorter in steep areas of the catchment and this definition of reaches was useful to characterise spatial variability in emissions as shown later. When estimating *k*_*600*_, the models were applied only in the velocity range covered by the *k* measurements i.e. up to a velocity of 0.7 m s^−1^. Only 2% of the days had an average reach velocity that was above this limit and during these time periods the reach specific maximum *k*_*600*_was used.

The mean modelled *k*_*600*_ for the entire stream network was 21.3 ± 64.8 m d^−1^ (median was 4.6 m d^−1^; *n*_modelled_ = 51133; Fig. S2). A few steep reaches in the stream network displayed very high *k*_*600*_ and thus skewing the mean *k*_*600*_for all reaches (Figs S3 and S4). The mean modelled *k*_*600*_ differed widely between locations and slope categories (General Linear Model (GLM), *p* < 0.001; Fig. S4; see Methods for categories) highlighting a large spatial variability. The mean *k*_*600*_ from location L7 (see Fig. 1), which has a steep elevation with waterfalls before entering lake Skottenesjön, was more than 3 times higher than the overall mean. Location L5, which represents a relatively flat area of the catchment with low water velocity, had the lowest mean *k*_*600*_ (Fig. S4). The slope category S5, including the steepest reaches, had 5 times higher mean *k*_*600*_ than the overall mean (Fig. S4; Table 3).

The modelled *k*_*600*_ values were also highly variable in time. The modelled mean *k*_*600*_ from the highest discharge periods (greater than four times the mean daily discharge) was more than 3 times higher than the overall mean modelled *k*_*600*_ from the two years (Table 3). In particular, flooding events in the reaches with higher slope could result in an up to 7-fold increase in *k*_*600*_ (Fig. S5). Moreover, there was an inter-annual variability in *k*_*600*_ with 1.4 times higher *k*_*600*_ in 2014 than in 2013 (Fig. S5).

### Spatio-temporal variability in CH_4_ concentrations

**Figure 3:**
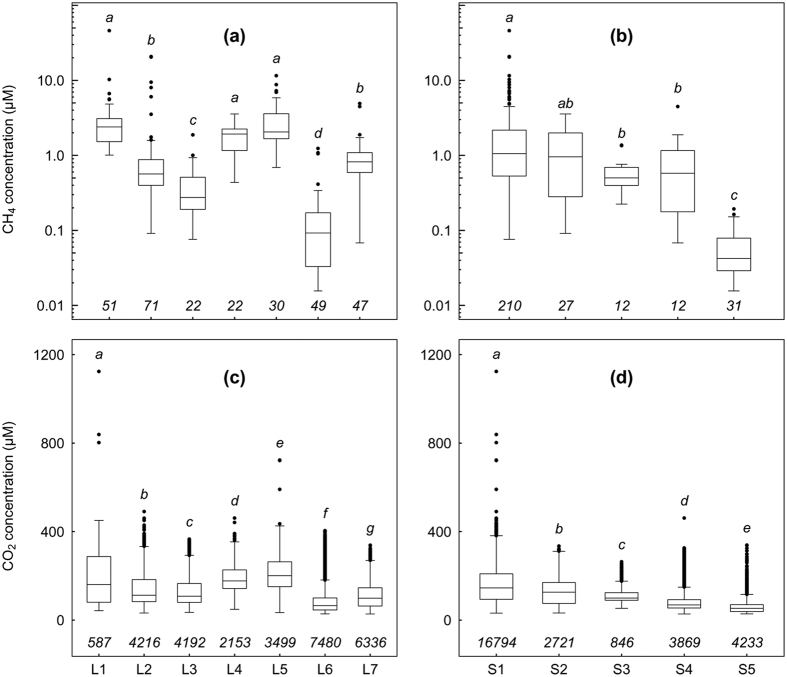
Boxplots of CH_4_ (**a**,**b**) and CO_2_ (**c**,**d**) concentrations measured in the streams grouped into location and slope categories. The boxes show quartiles and the median, the whiskers denote data within 1.5 times of the interquartile range and the black closed circles denote values outside the interquartile range. The letters above the boxes represent Tukey’s post-hoc test and boxes with different letters had significantly different concentrations (*p* < 0.05). The numbers below the boxes are the number of measurements in each category. Note the log_10_ scale in y-axis of panels (**a**,**b**). The concentrations in equilibrium with atmospheric concentrations are 0.004 μM and 23.5 μM for CH_4_ and CO_2_, respectively.

The stream water CH_4_ concentrations were highly variable and with a mean concentration of 1.7 ± 3.5 [0.01–46.1] μM (mean ± 1 SD [min − max]). For comparison, the theoretical CH_4_ concentration in equilibrium with the atmosphere (2.0 ± 0.09 ppm measured during the study) was 0.004 μM. Hence, CH_4_ was detected in all of our samples with concentrations that were more than 2 to over 11000 times supersaturated relative to atmospheric levels. The mean CH_4_ concentrations in the sampling points of L1, L4 and L5 were similar and higher than the mean stream concentration (GLM, *p* < 0.001; Fig. 3). The sampling points in L2 and L7 had similar concentrations near the overall mean. The samples from L6 showed the lowest CH_4_ concentrations (Fig. 3). The mean CH_4_ concentration in samples from the slope category S1 was significantly higher than S3, S4 and S5 but similar to S2 (Fig. 3). The mean concentration in S5, which had the highest water velocity, was lower than all other slope categories.

The CH_4_ concentrations showed large temporal variations (Fig. S6). However, CH_4_ concentrations had weak or no correlations with the variables investigated (water temperature, velocity, discharge and *k* values for CH_4_; all variables, measured at multiple locations in space, were normalised to the 2-year mean for each location to remove spatial variability when testing for temporal correlations).

### Spatio-temporal variability of CO_2_ concentrations

The mean stream water CO_2_ concentration was 131.6 ± 79.8 [range 27.5–1124.1] μM (corresponding to 2463 ± 1546 [520–22118] μatm when expressed as partial pressure of CO_2_). These concentrations correspond to excess concentrations of 1.3 to 54 times when compared to atmospheric levels (mean atmospheric CO_2_ concentration of 405 ± 28 ppm measured during the study). All our measurements showed that the streams were supersaturated relative to the atmosphere at all times. The CO_2_ concentrations occasionally reached higher than 15000 μatm (approx. >670 μM) especially close to the inlets and outlets of the lake Följesjön. In general, the spatial variability in CO_2_ was lower than for CH_4_ (Fig. 3). Location category L6 had the lowest mean CO_2_ (GLM; *p* < 0.001; Fig. 3). The mean CO_2_ concentrations in L1, L4 and L5 were 1.4, 1.4 and 1.6 times higher than the overall mean stream CO_2_ and the mean concentrations in L2 and L3 were close to the overall mean (Fig. 3). If instead organizing data based on slope category, the mean CO_2_ concentration in the slope category S1 was 1.2 times higher than the mean stream CO_2_ and, as for CH_4_, the CO_2_ concentrations were lowest in slope category S5 (Fig. 3). Local measurements upstream and downstream of steep stream sections/waterfalls showed that CO_2_ concentrations were consistently lower downstream due to the high turbulence causing a rapid loss of CO_2_ over a short distance (see Fig. S7 for an example).

The temporal variability in CO_2_ concentration was complex (Figs S6 and S7) and no clear relationships with discharge were found. High discharges increased the CO_2_ in some periods, but not consistently. During the dry periods, small pools of standing water were formed in the streams and had a relatively high CO_2_. High discharges after these dry periods sometimes diluted the CO_2_.

Biweekly CO_2_ and CH_4_ concentrations were not correlated in time (after normalisation to remove spatial variability; Pearson’s *r* = −0.054, *p* = 0.57). Diel variability in CO_2_ concentration was frequently observed from the high resolution CO_2_ sensor data (Fig. S7). CO_2_ concentrations had weak or no correlations with the tested variables water temperature, velocity, discharge and *k* values for CO_2_ (all variables measured at multiple locations were normalised as in the corresponding test for CH_4_ described above).

### Emissions of CH_4_ and CO_2_

**Figure 4:**
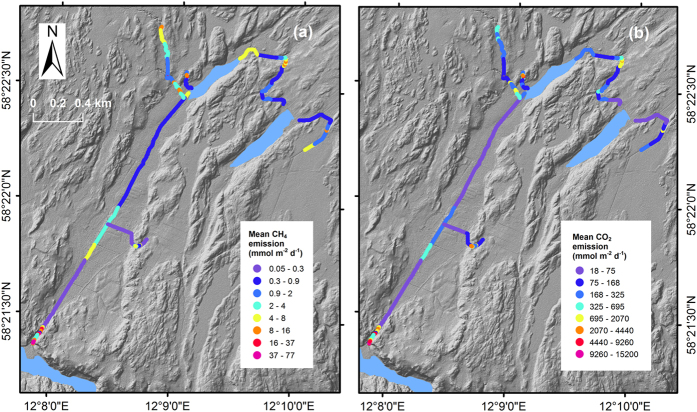
Mean modelled CH_4_ (**a**) and CO_2_ (**b**) emissions from the streams of SRC showing hotspots for emission. The majority of the streams had low emissions, but some reaches had high emissions due to high *k* or high concentrations or a combination of both. The figure was created with ArcMap 10.3.1 available from http://www.esri.com/. The background maps, obtained from Lantmäteriet (National Land Survey of Sweden), were published under the copyright agreement i2012/898 with Linköping University.

The mean CH_4_ emission was 8.8 ± 23.5 [0.009–930] mmol m^−2^ d^−1^ (standard deviation and range includes all values, i.e. both spatial and temporal variability; *n*_modelled_ = 52332; uncertainty range for mean 3.5 to 14.0 mmol m^−2^ d^−1^; assuming an uncertainty of ±60% of mean, see Methods). The mean CO_2_ emission was 1600 ± 4800 [3.3–90300] mmol m^−2^ d^−1^ (*n*_modelled_ = 52332; uncertainty range for mean 1200 to 2000 mmol m^−2^ d^−1^; assuming an uncertainty of ±25% of mean) from all sections (Figs 4 and S4). At location L7, which had the highest mean *k*_*600*_, the mean CH_4_ and CO_2_ emissions were 3 times higher than the overall mean (Figs S3 and S4). The lowest mean CH_4_ emission was observed for L6, which had the lowest CH_4_ concentrations, and L5, which had the lowest *k*_*600*_ (Figs 3, 4 and S4). L5 also had the lowest mean CO_2_ emission (Fig. S4).

The calculated emissions from the gas concentrations and the modelled *k*_*600*_ showed good relative agreement with independent measurements used for validation, including mass balance calculations for steep sections from upstream and downstream differences in gas concentrations, and drifting flux chamber emission measurements in flat areas (see Supplementary Information; Table S1 and Fig. S8).

**Figure 5:**
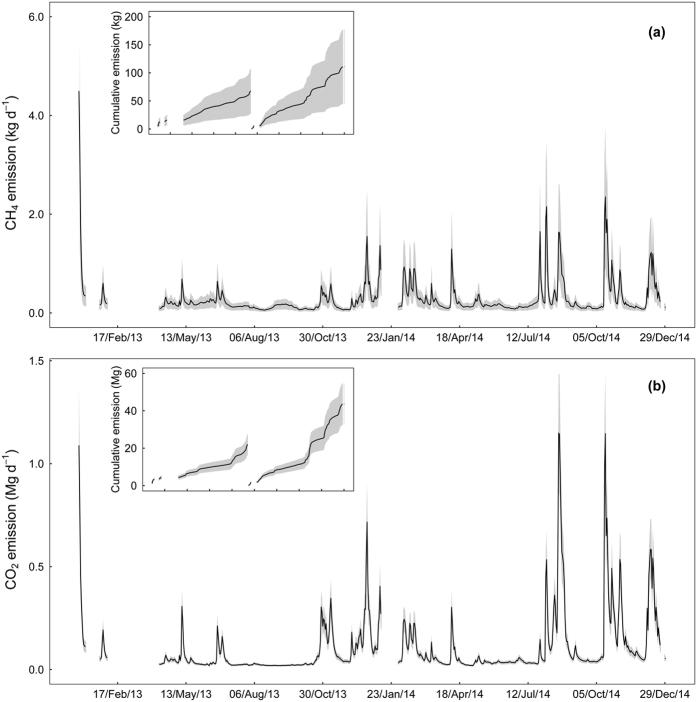
Daily emissions of CH_4_ (**a**) CO_2_ (**b**) from the studied streams in the two years. The inset panels in (**a**,**b**) shows the cumulative emissions of the two gases for the corresponding period. The shaded region represents an assumed uncertainty of ±60 and 25% of mean for CH_4_ and CO_2_ emissions, respectively (see Methods for details).

Large temporal variability in emissions of CH_4_ and CO_2_ were noted (Fig. 5). Stepwise multiple linear regressions of modelled emissions with gas concentrations or *k* together with all the slope/location categories showed that the CO_2_ emissions were largely driven by *k* as *k* for CO_2_ alone explained 83% of the variations in the emissions over time (*p* < 0.001). This was very different compared to the CH_4_ emissions, for which *k* for CH_4_ explained only 50% of the temporal variation (*p* < 0.001). The CH_4_ concentrations were a better predictor for CH_4_ emissions, as they explained 72% of the variability together with slope categories (*p* < 0.001). The cumulative CH_4_ and CO_2_ emissions from each of the sections showed higher increments in emissions during high discharge periods (Fig. 5). This pattern was clearer in 2014 and the total annual emissions of CH_4_ and CO_2_ in 2014 were 1.6 and 2 times higher than in 2013, respectively (Fig. 5).

## Discussion

### Patterns in gas transfer velocity

High variability in *k*_*600*_among stream sections was observed with very high values for steep sections including small water falls. The mean measured *k*_*600*_ from all reaches measured was 67.5 m d^−1^ while the mean *k*_*600*_ excluding the high turbulent reaches (B, C and F) was 6.5 m d^−1^. The mean modelled *k*_*600*_ for the entire network, accounting for the relative area distribution as 90.5% of the stream area in the catchment has a slope less than 1% (Table 3), was 21.3 m d^−1^. Humborg *et al*.^32^ estimated *k*_*600*_for Swedish streams of stream orders 1 to 6 ranging from 6.3 to 15.5 m d^−1^ using equations from O’Connor and Dobbins^33^. Campeau *et al*.^12^ measured mean *k*_*600*_ values in the range of 0.6 to 2.3 m d^−1^ in the rivers and streams of a relatively flat landscape in Northern Québec, Canada. Crawford *et al*.^34^ reported *k* for CO_2_ ranging from 0.3 to 13.5 m d^−1^ in the Northern Highlands Lake District, USA. Some other studies, for example Billett and Harvey^27^, measured high gas transfer velocities in headwater peatland streams in the UK with a median and mean *k* of 16.1 and 41.0 m d^−1^, respectively. Similarly, Butman and Raymond^35^ modelled a mean *k* of 18.0 m d^−1^ for streams and rivers in the relatively steep landscape of western USA. Extreme *k*_*600*_ values in the range of 864 to 1848 m d^−1^ were measured in steep rapids of Colorado River, Grand Canyon, USA^36^. Thus, our *k*_*600*_ values are largely within the ranges previously estimated in studies of stream reaches having highly different characteristics.

Several models to predict *k*_*600*_ from e.g. stream slope and water velocity have been published. When testing if seven such models^15^ could reproduce our measured *k*_*600*_ values, we found good correspondence at *k*_*600*_ levels below 100 m d^−1^. However, at higher *k*_*600*_ levels the models substantially underestimated our measured values (modelled *k*_*600*_ was 20–45% of measured *k*_*600*_ in the highest range). This could be explained if the previously published models were not based on data for high slope stream sections and therefore not calibrated or validated for high *k* situations.

### Gas concentrations

The range in CH_4_ concentrations measured in our study (0.01–46.1 μM; mean–1.7 μM) was within the ranges reported for streams in the literature. Our CH_4_ concentrations were higher than the range (0.2–5.8 μM) measured in the Stordalen catchment, Northern Sweden^9^ and in a first order stream (0.04–0.1 μM) in Tennessee, USA^11^, but similar to the mean concentrations of 0.05 up to 1.9 μM measured in headwater streams of Scotland^37^. Similar CH_4_ concentrations were also reported in studies in boreal region of Northern Québec, Canada (0.04–49.2 μM)^12^, in headwater streams of U.K. (0.002–15.3 μM)^27^ and in extensive sampling of headwaters in south Sweden (0.16–57.4 μM)^13^. The range in CO_2_ concentrations measured in our study (27.5–1124.1 μM) was similar to the range (23.8–714.7 μM) previously reported in many studies in Northern Québec, Canada, U.K. and Sweden^9^,^12^,^13^,^27^.

The gas concentrations in stream water represents a net balance between (1) the emission rates from the stream and (2) the input from terrestrial and aquatic sources along the stream network^5^,^13^,18–20,37. The observed variability in concentrations seemed associated with both these factors. Locations typically surrounded by organic soils (L1, L4 & L5) had higher concentrations which agrees with previous findings^5^,^19^,^20^,^37^,^38^,^39^, indicating the importance of terrestrial and wetland gas input. At the same time, low gas concentrations in the high slope reaches indicates that local concentrations within areas with similar input can be regulated by differences in gas loss rates. Given this complex regulation of concentrations for both gases, high resolution monitoring with adequate coverage of spatial variability is needed to develop more accurate modelling of stream CH_4_ and CO_2_ concentrations^13^,^32^.

### CH_4_ and CO_2_ emissions from the studied streams

Previous measurements show a wide range of emissions from streams. CH_4_ emissions in the range of 0.03 to 0.8 mmol m^−2^ d^−1^ were measured in first order streams in Tennessee, USA^11^. CH_4_ emissions ranging from 2.2 to 37.4 mmol m^−2^ d^−1^ in headwaters in Scotland, 0 to 60.1 mmol m^−2^ d^−1^ in small streams in USA, and emissions of 0.8 to 5.0 mmol m^−2^ d^−1^ in headwaters of Alaska have been measured^18^,^34^,^37^. Lundin *et al*.^9^ reported mean CH_4_ emissions of 15.8 mmol m^−2^ d^−1^ in streams of Northern Sweden. CO_2_ emissions from headwater streams of UK and streams of central Germany ranged between 316.1 and 3461.0 mmol m^−2^ d^−1 ^27, and from 23.0 to 355.0 mmol m^−2^ d^−1^, respectively^40^. Others such as Hope *et al*.^37^ and Crawford *et al*.^34^ have estimated emissions in the range of 21.6 to 3820.0 mmol m^−2^ d^−1^ and −50.0 to 2030.0 mmol m^−2^ d^−1^, respectively. Mean CO_2_ emissions of 1300.0 mmol m^−2^ d^−1^ were reported in streams of Northern Sweden by Lundin *et al*.^9^. Our emissions (mean CH_4_ emission of 8.8 ± 23.5 and mean CO_2_ emission of 1600 ± 4800 mmol m^−2^ d^−1^) were within previously measured ranges, but on the high side which is explained by the organic rich soils in the catchment and that we tried to account for the full spatio-temporal variability in emissions including high turbulent sections of the stream network, which was important for the total emissions (discussed further below).

The CH_4_ emissions reported includes the transport of dissolved CH_4_ across the water-air boundary layer (sometimes called diffusive emission). Enhanced emissions of CH_4_ via microbubbles have recently been suggested^41^. At the concentration levels found in this study CH_4_ microbubbles would not form spontaneously, i.e. although there was a supersaturation relative to 2 ppm CH_4_ in the air, there was not a supersaturation relative to 100% CH_4_. Rapid degassing of both CH_4_ and CO_2_ into small air bubbles formed in the highly turbulent (e.g. waterfall sections) were integrated in the *k* values measured, and thereby included in our model. Release of CH_4_ bubbles from sediments could only have been captured by the drifting chambers used for model validation but the limited extent in time of these measurements implies that ebullition was not representatively measured. Hence, overall CH_4_ emissions may have been underestimated because ebullition was not accounted for.

The general agreement between the three independent methods to estimate emissions–(*i*) modelling from *k* determined with propane tracer injections combined with water concentrations measurements, (*ii*) mass balance calculations along selected stream sections, and (*iii*) drifting chambers –in spite of highly variable conditions along the stream network, indicates that our measurements and model results are relatively robust (see Supplementary Information; Table S1 and Fig. S8). Approaches (*i*) and (*ii*) should be comparable because both are based on mass balance calculations of gas loss over short stream sections for either the injected propane tracer or in this case CO_2_ (which dominates the dissolved inorganic carbon pool in the studied streams). Lower correspondence is expected between approach *(i)* and *(iii)* because drifting chambers measure gas exchange along a specific path of the stream. Additional questions regarding if drifting chambers disturb the water surface have been raised^43^, but recent results show that the chamber type used here is suitable for gas flux measurements in both lakes and streams^44^,^45^. Overall, given the highly variable conditions along streams, careful validation using independent but comparable measurement approaches is recommended.

The variability in emissions was primarily driven by the spatial variability in *k* (i.e. stream slope and water velocity) and gas concentrations regarding both studied gases. *k* was relatively more important for CO_2_ emissions, while CH_4_ emissions seem to be more dependent on the water CH_4_ concentrations. This was likely due to the higher variability in CH_4_ concentrations making emissions more dependent on concentrations than on *k*. These differences are important for designing future efforts to better estimate stream emissions, and optimising the resource allocation to *k* versus concentration monitoring. However, at the local level, both *k* and concentrations were influenced by discharge with gas import and export. Hence, the difference among years related to discharge is expected and was also noted: discharge in 2014 was 1.7 times higher than in 2013 being consistent with the different emission estimates among the years (Fig. 5).

The total emissions of CH_4_ and CO_2_ from the studied stream network were estimated to be 89.5 (36.0–143.1) kg yr^−1^ and 32.9 (24.6–41.2) Mg yr^−1^, respectively, from an area of 6319 m^2^ (see Supplementary Information for stream area estimation). In terms of CO_2_ equivalents, this was 35.4 (25.6–45.2) Mg yr^−1^ (when assuming a warming potential of CH_4_ of 28 over a 100-yr horizon according to Myhre *et al*.^46^), with CH_4_ contributing 7%. Previous stream studies have suggested that CH_4_ emissions are minor when compared to CO_2_^27^,^38^. However, Crawford *et al*.^34^ and Campeau *et al*.^12^ reported that CH_4_ accounted for 26 and 34% of the total CO_2_ equivalents emitted from streams. Our study indicates that CH_4_ can contribute substantial CO_2_ equivalent emissions from streams, and that the large spatial variability in CH_4_ emissions needs consideration in future studies.

### The importance of spatio-temporal variability for total emissions

An analysis of emissions from different slope and discharge categories revealed high emissions in high slope reaches and during high discharge periods (Table 3; Figs 5 and S4). The streams with a slope <1% occupied 90% of the total stream surface area but their mean emissions of CH_4_ and CO_2_ emissions per m^2^ were 5 times lower than the overall mean (Table 3). The slope categories S3, S4 and S5 had emissions higher than the overall mean. The slope category S5, which occupied just 0.9% of the total stream surface area, had areal emissions that were 3 and 4 times higher than the overall mean emissions for CH_4_ and CO_2_, respectively (Table 3). Therefore, despite the small areal coverage, the slope categories S4 and S5 (which occupied less than 2% of the total steam area), contributed to 18 and 30% of total CH_4_ and CO_2_ emissions, respectively. However, it has to be noted that our modelled emissions are conservative and underestimated fluxes from highly turbulent/water fall sections (see Supplementary Information and Fig. S8).

Regarding temporal variability, 69% of the days had a stream discharge that was lower or equal to the mean discharge of the two-year study period, but the emissions from this period were lower than the overall mean CH_4_ and CO_2_ emissions (Table 3). The mean emissions steadily increased as the discharge increased and clearly showed highest emissions during high discharge periods. For example, during days with a discharge greater than 4 times the mean daily discharge, representing 5% of the study period, the mean emissions of CH_4_ and CO_2_ were more than 3 times the overall mean emissions (Table 3). The high discharge periods, with discharge greater than 3 times the mean daily discharge, occurred less than 10% of the study period, but were responsible for 37 and 43% of total CH_4_ and CO_2_ emissions, respectively. Had we sampled in more or less flat areas and during moderate flows (ignoring the extremes in both cases), we still could have covered majority of the space and time (90% of area and 69% of time) but arrived at substantially underestimated emissions i.e. the emissions from flat areas and moderate flows were just 55 and 36% of CH_4_ emissions and 41 and 26% of CO_2_ emissions, respectively. This illustrates the importance of representative sampling and modelling when scaling to the landscape level. Hotspots of degassing along the stream network and high discharge events should be taken into account when possible. Moreover, *k* values from small areas with high water turbulence should not be extrapolated to large areas because this lead to overestimated emissions.

Given these results, accurate assessments of gas emissions from stream networks depend on consideration of the large spatial variability in both *k* and concentrations. Although steep sections can have low gas concentrations, their high *k* can make these sections important for emissions. On a temporal scale, high discharge events are important and a large of part of the annual emissions may happen during these very short periods. Projected shifts in the hydrological regimes for many regions, as a result of a changing climate, may contribute further to the temporal patterns. The potential importance of the short high discharge periods has been discussed previously, but quantifications of their relative importance are still rare. We conclude that further development of sensor network based measurements and the types of modelling approaches presented here would be beneficial to better account for the hot spots and hot moments in future gas emission assessments.

## Methods

### Study site

The study was conducted in the Skogaryd Research Catchment (SRC; 58°22′N, 12°9′E), situated in Southwest Sweden (Fig. 1). The 7 km^2^ catchment is mostly covered by forest (58% coniferous forests, 14% mixed forest), dominated by *Picea abies* (L.) H. Karst. and *Pinus sylvestris* L., and to a minor part by agricultural land (9%). About 14% is covered by cleared forest, 4% by mire and 1% by lakes and streams. The annual mean temperature and precipitation were 7.0 °C and 910 mm, respectively, for the years 1983–2013 (Swedish Meteorological and Hydrological Institute; http://luftwebb.smhi.se/). The altitudes in the catchment ranged from 51 to 78 m above the average sea level. The main stem of the stream network originates in a mire just upstream of Lake Erssjön (Fig. 1). Via a chain of streams and lakes (Lake Erssjön and Lake Följesjön) the main stem drains into the large Lake Skottenesjön (Fig. 1). The network consists of a mixture of flat and slow-flowing areas, especially between the outlet of Lake Följesjön to Lake Skottenesjön, whereas other parts of the network flow through relatively steep terrain creating small waterfalls and turbulent conditions. The majority of the stream network is affected by man-made ditching conducted 100–150 years ago to improve forest and agricultural productivity.

### Measurements of gas transfer velocity (*k*)

Gas transfer coefficients (*g*) were determined using injections of a volatile gas tracer, propane (C_3_H_8_), as described in similar studies^11^,^14^,^37^,^47^ and in the Supplementary Information. Six stream reaches ranging in length from 20 to 54 m (Fig. 1 and Table 1) were chosen to represent end-members in morphological conditions of the streams within the catchment. The reaches A, D and E are straight ditches with gentle slope (<1.5%) whereas the reaches B, C and F represent relatively steep (>7.5%) and highly turbulent stream sections (Fig. 1 and Table 1).

The gas exchange coefficients of propane (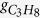) from the tracer injections were calculated according to Genereux and Hemond^48^ using the modification suggested by Wallin *et al*.^14^ to correct for the dilution by groundwater inputs along the study reach according to


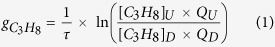


where 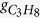 is the gas transfer coefficient of propane (min^−1^), *τ* is the reach travel time (min), [C_3_H_8_]_*U*_ and [C_3_H_8_]_*D*_are the relative concentrations of propane in the upstream and downstream samples, respectively, and Q_*U*_ and Q_*D*_ are the discharge (L s^−1^) at the upstream and downstream stations, respectively. The difference in the upstream and downstream discharge calculated from the estimated discharge (see below) ranged from 0.06 to 0.5%, and this was used to calculate Q_*U*_ and Q_*D*_from the measured mean discharge. The 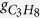
was converted to gas transfer velocities (*k*; m d^−1^) by multiplication with the average stream depth of the reach at each sampling occasion. An alternative approach to derive *k* from the general diffusive flux calculation^49^


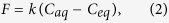


where F is the flux to the atmosphere–here determined as the loss of propane from a water section during the reach travel time, *C*_*aq*_ is the water concentrations as measured and *C*_*eq*_ is the theoretical water concentration in equilibrium with the atmospheric partial pressure according to Henry’s Law, yielded almost identical *k* values.

The *k* values for propane were converted to *k*_*600*_ (i.e. *k* for a gas having the Schmidt number of 600; this corresponds to the *k* for CO_2_ in freshwater at 20 °C) to enable comparison across temperatures and different gases according to Wanninkhof^50^ as


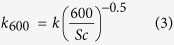


where *k* denotes the gas transfer velocity (m d^−1^) and *Sc* is the Schmidt number of the gas in focus according to Wanninkhof^31^. *Sc* for propane was calculated as described in Raymond *et al*.^15^. We found a discrepancy in temperature dependent diffusion coefficients for propane in the literature^51^,^52^, in turn leading to alternative Schmidt numbers having a clear impact on the outcome of the emission calculations. We used the diffusion coefficients from Wise and Houghton^52^ which were determined in a relevant temperature interval, have been widely used previously, and resulted in the best fit between our emission model and the independent gas emission measurements used for validation (Figs 2 and S8).

The *k*_*600*_ values were converted to gas and temperature specific *k*-values for CO_2_ and CH_4_ according to


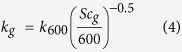


where *k*_*g*_ and *Sc*_*g*_ denote *k* and Sc for a specific gas (here CO_2_ or CH_4_) at a specific temperature (the water temperature during the time period in focus). Stream velocity (m s^−1^) for each tracer injection was calculated by dividing the length of the reach by the reach travel time (see Supplementary Information).

### Measurements of stream water CH_4_ and CO_2_ concentrations

#### CO_2_ concentrations

The CO_2_ concentrations in the stream network were measured using plastic chambers equipped with CO_2_ sensors (CO_2_ Engine^®^ ELG, SenseAir AB, Sweden; range 0–10000 ppm) described in detail in Bastviken *et al*.^53^ (see Supplementary Information for details). In 2013, 8 chambers were used at different locations in the stream network (Fig. 1). Due to practical constraints in using chambers for long-term deployments (rapid changes in water level, dry season with little water, storm events with rapid water flow etc.) there were data gaps. In 2014, 20 chambers were used to obtain a better spatial representation. The data from the sensors were filtered to remove condensation peaks^53^ by removing values greater than 20% of 12 hour averages before and after each measurement. A total of 28,463 CO_2_ measurements were made in the two years.

#### CH_4_ measurements

The CH_4_ concentrations were determined by a headspace equilibration method^54^ (see Supplementary Information for a description). The concentrations of CH_4_ in the stream water were measured approximately every two weeks in 2013 (May-Nov) and 2014 (Apr-Nov) at four monitoring stations whose locations are given in Fig. 1. In 2014, CH_4_ concentrations were also measured close to the CO_2_ chamber measurements to better cover the spatial variability. CH_4_ concentrations measured during the propane injections were included in the data analysis. A total of 292 stream water CH_4_ concentration measurements were conducted during the study period. Additional details on gas analysis using a gas chromatograph are given in the Supplementary Information.

### Stream network *k* estimates

A digital elevation model (DEM) of the catchment with a 2 m horizontal resolution was obtained from Lantmäteriet (https://maps.slu.se/get/). The stream network of the catchment and the overall and partial drainage areas were extracted from the DEM using the Spatial Analyst toolbox in ArcGIS 10.3. The daily discharge for each 2 m stream section, out of the, in total, 6.4 km long stream network was estimated as follows: (1) Stream discharge was measured at four monitoring stations in the catchment according to the methodology described by Wallin *et al*.^55^ (see also Supplementary Information). From these measurements we generated daily average discharge at four locations in the stream network. (2) The upstream drainage area for each of the four stations were determined using the Spatial Analyst toolbox in ArcGIS 10.3. (3) Hence, for each day we could make a regression equation, *D* = *bA*, where *D* is discharge, *A* is upstream drainage area, and *b* is a constant (i.e., the average discharge per unit area), based on the discharge data for the specific day from the four stations. The study encompassed 623 days (January 2013 to December 2014, except during periods when average air temperatures were below 0 °C for more than 3 days in a row and discharge measurements indicated that streams were frozen), meaning that 623 separate such equations valid for their respective day were made. R^2^ exceeded 0.92 for all days (average 0.98). The range of b was 0.2 to 315. (4) The upstream drainage area for each 2 m stream section was determined from ArcGIS and was multiplied with *b* of the 623 equations to estimate average daily discharge for each such 2 m section.

The stream network was divided into 84 reaches where the length of each reach was defined by an elevation difference of 0.5 m. The slopes (%) of the stream reaches were calculated by dividing the elevation difference of each reach by the reach length.

We established a regression model with velocity as a function of discharge and slope measured in the stream reaches where tracer injections were conducted (Table 2). This model was used to extrapolate the velocity for all 84 stream reaches from January 2013 to December 2014 using daily reach average discharge and slope for each individual stream reach. *k*_*600*_ values in the whole stream network were then modelled from stream slope and water velocity as predictor variables (see Fig. S9 for a brief summary of methods).

### Emission estimates for the stream network

Emissions were calculated by using Eq. 2. Daily emissions were calculated for each of the 84 reaches, using reach specific daily *k* estimates. No good relationship between CH_4_ and CO_2_ concentrations and any independent variables were found (see Results), thus modelling spatially distributed CH_4_ and CO_2_ concentrations at a daily time step was not possible. Instead, CO_2_ and CH_4_ concentrations in the un-sampled reaches were estimated, based on manual concentration measurements from 20 points of the stream network (measurements done on 5 occasions for CH_4_ and 7 for CO_2_ in 2014). Concentrations were interpolated between these 20 points (e.g. assuming linear change in concentration between upstream and downstream measurements when available). The ratios of the interpolated concentrations in relation to the concentrations at the most frequently samples measurement points of CH_4_ and CO_2_ were then calculated, yielding relative concentrations in each stream reach. Assuming that these relative concentrations were valid for the time periods between the spatially resolved samplings, the concentration levels in each reach over time were estimated from the concentrations at the temporally resolved sampling points (biweekly for CH_4_ and daily mean for CO_2_). For the days between sampling occasions, concentrations were linearly interpolated to gap-fill. Daily emissions for each of the 84 reaches were then calculated based on the stream surface area of each reach (expressed in kg d^−1^ for CH_4_ and Mg d^−1^ for CO_2_). The emissions from all 84 reaches were then summed and total emissions of CH_4_ and CO_2_ from the streams were expressed in kg yr^−1^ or Mg yr^−1^. By far the largest uncertainty in the emission estimates come from the relative concentration ratios used to represent spatial differences along the streams during the different periods to interpolate concentrations within the stream network. Therefore, the coefficient of variations (CV) of the interpolation ratios of each reach were calculated and the maximum CV was used to estimate uncertainty range for the mean for all reaches, which were 60 and 25% for CH_4_ and CO_2_ emissions, respectively.

### Data analyses

Residuals in the linear regressions were checked for normal distribution and randomness of errors and the original variables were log_10_ transformed when this criterion was not met. The predicted values from the log_10_ transformed linear regression equations were back transformed to original units using the correction proposed by Newman^56^. The concentrations, *k*_*600*_ and emissions for CO_2_ and CH_4_ were divided into seven groups based on location (L1 to L7; referred to as location categories; see Fig. 1) and into five groups based on slope (S1 (0–1%), S2 (1–2%), S3 (2–4%), S4 (4–6%) and S5 (6–21%); referred to as slope categories). For CH_4_ and CO_2_ concentrations, slope categories were based on the slope of the reach where the measurement points were located. A univariate General Linear Model (GLM) with Tukey’s post hoc test was used, with location and slope categories as factors, to analyse spatial variability. All statistical analyses were done in IBM SPSS Statistics 23 (IBM Corp., USA) with a significance level of 0.05. GIS related analyses were performed in ArcGIS 10.3 (Esri Inc., USA).

## Additional Information

**How to cite this article**: Natchimuthu, S. *et al*. Spatio-temporal patterns of stream methane and carbon dioxide emissions in a hemiboreal catchment in Southwest Sweden. *Sci. Rep.* **7**, 39729; doi: 10.1038/srep39729 (2017).

**Publisher's note:** Springer Nature remains neutral with regard to jurisdictional claims in published maps and institutional affiliations.

## Supplementary Material

Supplementary Information

## Figures and Tables

**Table 1:** Stream characteristics given as the mean (or minimum–maximum) of physical parameters of the studied reaches and *k*_*600*_ measured using propane injections.

	A	B	C	D	E	F
Catchment area (km^2^)	0.6	1.5	0.4	0.5	0.4	0.5
Length (m)	34	32	54	31	20	24
Slope (%)	1.5	7.5	7.6	0.5	0.3	19.3
Mean width (m)^a^	0.9–1.1	0.8–2.4	0.7–0.9	0.5	0.7–1.3	0.7
Mean depth (m)^a^	0.08–0.2	0.09–0.4	0.09–0.2	0.2	0.1–0.4	0.1
Reach area (m^2^)^a^	31.6–40.5	24.4–77.6	37.4–46.7	16.8	13.4–28.2	16.9
Discharge (L s^−1^)	27.6 (6.5–101.2)	82.8 (5.2–274.7)	23.2 (8.5–58.4)	10.1	30.2 (4.5–117.4)	7.3
Reach travel time (min)	3.4 (1.5–5.1)	2.4 (0.7–5.7)	4.4 (1.9–6.4)	3.9	2.6 (0.8–4.0)	3.2
Mean velocity (m s^−1^)	0.2 (0.1–0.4)	0.3 (0.09–0.7)	0.3 (0.1–0.5)	0.1	0.2 (0.08–0.4)	0.1^b^
Water temperature (°C)	9.2 (5.5–15.2)	9.7 (5.6–17.2)	8.4 (6.8–12.9)	5.8	8.4 (5.2–13.3)	6.4
*k*_*600*_ (m d^−1^)	10.7 (2.6–27.5)	152.6 (13.9–558.7)	72.5 (37.1–185.7)	3.0 (2.2–4.4)	1.2 (0.2–3.5)	73.7 (71.7–75.3)
*n*^c^	12 (5)	15 (5)	12 (4)	3 (1)	8 (5)	3 (1)

^a^Range of mean width, depth and area of the reaches, except for reaches D and F where measurements were done once.

^b^Although the slope in reach F was the highest, on the measurement day the discharge was very low and thus generated low velocity value.

^c^Number of observations of *k*_*600*_; the number of propane injections in each reach is given in the brackets (reaches D and F were sampled once).

**Table 2:** Regression equations predicting stream velocity (V, in m s^−1^) from discharge (D, in L s^−1^) and slope (S, in %), and *k*_*600*_ (m d^−1^) from stream velocity and slope.

Model no.	Regression equation	*n*	Adjusted R^2^	*p*	MSE^a^
1	Log_10_V = −1.323 + (0.466 × log_10_ D) + (0.056 × log_10_S)	21	0.91	<0.001	0.006
2	Log_10_ *k*_*600*_ = 0.319 + (2.110 × V) + (1.026 × log_10_S)	53	0.92	<0.001	0.050

^a^Mean square error of the regression.

**Table 3:** Ratio of mean emissions and *k* from different slope and discharge categories to the overall mean values indicating under or overestimates if only a single slope and discharge category is considered.

Slope category^a^	Percent area (%)^b^	CH_4_ emission ratio^c^	CO_2_ emission ratio	*k* ratio
S1	90.5	0.2	0.2	0.1
S2	5.2	0.7	0.4	0.3
S3	2.6	1.5	1.4	1.0
S4	0.7	1.9	2.3	1.7
S5	0.9	3.1	3.8	5.1
**Discharge ratio**^d^	**Percent occurrence (%)**^e^	**CH**_**4**_ **emission ratio**	**CO**_**2**_ **emission ratio**	***k*** **ratio**
<1	69.0	0.6	0.5	0.5
1 to 2	15.7	1.2	1.4	1.5
2 to 3	5.9	2.0	2.4	2.6
3 to 4	4.2	2.4	2.7	2.8
>4	5.1	3.2	4.0	3.3

Ratios <1 denote underestimate and >1 denote overestimate.

^a^Slope of reaches divided into five categories; S1 (0–1%), S2 (1–2%), S3 (2–4%), S4 (4–6%) and S5 (6–21%).

^b^Mean percentage of area of each slope category.

^c^Emission estimates relative to whole-catchment mean; for example, a ratio of 1.5 means that the emission from the particular slope or discharge category was 1.5 times higher than the whole-catchment mean.

^d^Ratio of discharge to the mean reach discharge from each reach was calculated and divided into five categories for comparison.

^e^Percentage of time with the corresponding discharge ratios during the study period.
