# Supplementary material for: Spatio-temporal patterns of stream methane and carbon dioxide emissions in a hemiboreal catchment in Southwest Sweden
Source: Sci Rep. 2017 Jan 3;7:39729. doi: 10.1038/srep39729 (PMC5206626; doi:10.1038/srep39729)
Supplement: Supplementary Information [file srep39729-s1.pdf]

Supplementary Information for

Spatio-temporal patterns of stream methane and carbon dioxide emissions in a hemiboreal catchment in Southwest Sweden

Sivakiruthika Natchimuthu[1*], Marcus B. Wallin[2,3], Leif Klemedtsson[4], David Bastviken[1]

[1]Department of Thematic Studies – Environmental Change, Linköping University, 581 83 Linköping, Sweden

[2]Department of Earth Sciences, Uppsala University, 752 36 Uppsala, Sweden

[3]Department of Ecology and Genetics/Limnology, Uppsala University, 752 36 Uppsala, Sweden

[4]Department of Earth Sciences, University of Gothenburg, 405 30 Gothenburg, Sweden

*Corresponding author. Contact: +46 13 28 29 59, E-mail: sivakiruthika.natchimuthu@liu.se

Contents of this file

Supplementary Methods

Supplementary Notes

Supplementary References

Figures S1 to S9

Table S1

Supplementary methods

The propane injection method to measure *k*

Determination of *k* was done using a tracer gas approach previously used in similar types of stream systems [1-4]. Before the tracer gas injection, a pulse injection of a conservative tracer (NaCl) was made to measure the discharge (Q) and reach travel time (τ) throughout the stream reach. Two conductivity probes, (U24-001, Onset Computer Corporation, U.S.A.) set to log every 2 s, were placed at the upstream and downstream stations. Reach travel time was calculated by subtracting the times at which maximum electrical conductivity (EC) was reached at the two stations (this is the time taken for the water mass to travel from the upstream to downstream station). Using a 0.6 m long fine perforated tube, fine bubbles of propane (from 10 kg cylinders; AGA, Sweden) were injected at a constant rate into the streams at ~10 m upstream of the reach for 15-30 minutes prior to sampling to ensure proper mixing of propane within the reach and to attain steady state conditions. To sample the same water mass, water samples were collected at the upstream and downstream stations using a separation in time according to the reach travel time. Water samples of 5 mL were collected 2-3 cm below the surface and were injected into 20 mL vials that were prefilled with 100 µL of H_3PO_4 and pre-flushed with N_2 (sampling details as described below for CH_4 and CO_2 concentration measurements). The sampling procedure was repeated three times (2 samples each time × 3 replicates) at five minute intervals. Also, depth and width of the stream reach (every 1 or 2 m) were measured at each tracer injection (Table 1).

CO_2 concentration measurements

Due to the turbulence in the streams, the CO_2 inside the chamber headspace soon attains equilibrium with the water phase (in approximately 5 hours and not more than 24 hours at very slow moving reaches [5]). The CO_2 sensors (see Methods) were attached to the inside of

the chambers and driven by 9V batteries. They were calibrated in CO_2 free atmosphere (nitrogen gas) according to the guidelines by the manufacturer and set to log every 1 hour (see Bastviken *et al.* [5] for further details on construction and deployment). The chambers were checked every 2 - 4 weeks to replace batteries, download data, and for maintenance. Manual CO_2 measurements of the chamber headspace were analysed on a gas chromatograph every time the chambers were visited, to check and if needed correct for drift in sensor measurements. The sensor CO_2 was proportional to the pCO_{2aq} (the partial pressure of CO_2 in equilibration with the water concentration) according to Henry's law and was calculated as,

$$pCO_{2aq} = P_{total} \times \frac{CO_{2sensor}}{10^6} \qquad \text{(S1)}$$

where P_{total} is the air pressure (µatm), $CO_{2_{sensor}}$ is the CO_2 measured in the sensor (ppm). This pCO_{2aq} was converted to CO_2 concentrations (µM) by multiplying with temperature adjusted Henry's law constants [6].

CH_4 concentration measurements

For CH_4 concentrations, 60 mL of bubble free stream water was collected using a 60 mL syringe (Becton-Dickinson, U.S.A.) and injected into 100 mL glass vials capped with thick 10 mm bromobutyl rubber stoppers and sealed with aluminium caps (ApodanNordic, Denmark). The vials were pre-flushed with nitrogen, filled to overpressure and prefilled with 200 µL of 85% H_3PO_4. Before injecting water, the overpressures in the vials were released to equilibrate the pressure inside the vials with that of the atmosphere. The presence of acid in the vials reduced the pH of stream water to < 2, shown to inhibit CH_4 oxidation [7] and thus preserving samples until analysis. By analysing the vial headspace and using the ideal gas law, the number of moles of CH_4 in the headspace was determined. The number of moles of CH_4 dissolved in the water phase was determined using Henry's law adjusted for temperature [8].

The total number of moles from the gas phase and water phase were then divided by the volume of water to obtain CH_4 concentration in the stream water.

Gas analyses

CH_4, CO_2 and C_3H_8 samples were analysed using a gas chromatograph (7890A, Agilent Technologies, U.S.A.) with a 1.8 m × 3.175 mm Porapak Q 80/100 column from Supelco, a Flame Ionization Detector (FID) and a Thermal Conductivity Detector (TCD). Standards prepared by serial dilution of certified high concentration standards (5000 ± 100 ppm CH_4 and 50000 ± 1000 ppm CO_2) and independent certified standards of 9.97 ± 0.2 ppm CH_4 and 1985 ± 40 ppm CO_2 were used for calibration.

Velocity estimation

The relationship of stream velocity with discharge and slope measured at the sampled reaches had an adjusted R^2 of 0.91 ($p < 0.001$; see Table 2 for regression equations). The resulting model fitted well with the measured velocity data ($R^2 = 0.94$; $p < 0.001$; Fig. 2a) and therefore this relationship was used to estimate velocity for all the 84 reaches in the stream network. The mean of the modelled velocity was 0.2 ± 0.2 [0.01 – 2.0] m s^{-1} (median = 0.1 m s^{-1}, $n_{modelled} = 52332$; Fig. S2).

Validation of emission estimates

To validate low emissions obtained from the models, a few CO_2 emission measurements (n = 9) were performed at low flow conditions in the streams by using chambers fitted with CO_2 sensors (see Methods). The chambers were either drifted along the stream when the current was strong enough or they were placed in standing pools of water during summer. The logging interval were set from 30 to 120 s depending on the method (smaller interval for drifting chambers) and at least 5 measurement points were obtained. Rate of change in

concentration was obtained using linear regression and converted to moles using the ideal gas law. Emissions were calculated by dividing moles by area of the chamber and time. A comparison of emissions from both methods showed that the model results produced similar ranges as the emissions from chambers (Table S1).

To validate high emissions obtained from the models, a mass balance approach was used to estimate CO_2 emissions from the final 300 m stream length close to the outlet of lake Skottenesjön, where the emissions were usually high due to the presence of waterfalls generating very turbulent conditions. Simultaneous CO_2 concentration measurements from the upstream and downstream point over 35 days between April to July 2013 were used to estimate the emissions. Mean emissions from the modelled data using *k* for the reaches that covers this 300 m length were compared against the mass balance method to check the performance of the model presented here. A plot of emissions from both methods correlated well and the R^2 was 0.97 ($p < 0.001$; Fig. S8). The emissions from the *k* model were found to underestimate emissions in the tested range (< 10000 mmol m^{-2} d^{-1}) below which majority of our emissions fall (only 3% of emissions above this range). This may partly be due to our decision to not model *k* values outside the range of the measurements (see main text) and using the maximum measured *k* values for all water velocities above 0.7 m s^{-1}. This makes our model conservative at high slope sections.

Other measurements

Discharge was measured in the four monitoring stations in 2013 and 2014 (Fig. 1) by measuring stage height using an ultrasound sensor (710, MJK Automation, Sweden) on installed flumes every 10 or 30 minutes, connected to an ISCO 6712 station (Teledyne ISCO, U.S.A.) [9]. Flumes were installed in three out of four monitoring stations, and the ultrasound sensor was mounted above a natural section in one station close to the catchment outlet. Site

specific stage-height-discharge rating curves, obtained from manual discharge measurements using a handheld Flow Tracker device (SonTek, San Diego, U.S.A.), were used to derive discharge values in the four monitoring stations. The water temperature was logged every 10 minutes in the station close to the catchment outlet using a Multi-Parameter water quality monitoring sonde (600R, YSI, U.S.A).

Supplementary notes

Characteristics of reaches sampled for *k*

The mean width and depth of the sampled stream reaches ranged from 0.5 to 2.4 m and from 0.08 to 0.4 m respectively (Table 1). The area of the stream reaches ranged from 16.8 to 77.6 m^2 and the average slope along the whole reach ranged from 0.3 to 19.3% (Table 1). The discharge measured at the sampled reaches ranged from 7.3 to 274.7 L s^{-1}, and the highest discharges in reaches A, B, C and E were measured on August 20, 2014 (see Fig. 1 for reach locations; reaches D and F were sampled only once in November 2014). Among the four reaches, the lowest mean discharge was measured in reach C and the highest in reach B (Table 1). The reach travel times ranged from 0.7 to 6.4 minutes and the lowest mean travel time (2.4 minutes) and highest mean water velocity (0.3 m s^{-1}) was observed in reach B. The water temperature during the sampling varied from 5.2 to 17.2 °C (Table 1). The median k_{600} in reach B was significantly higher than A, D and E (Kruskal Wallis, p = 0.003, 0.033, <0.001 respectively), but similar to C and F ($p > 0.05$). The median k_{600} in reach E was significantly lower than B, C and F (p < 0.001, 0.001, p = 0.008 respectively) but similar to A and D ($p >$ 0.05).

Stream reaches in the network

The length of the 84 reaches, defined by an elevation difference of 0.5 m, ranged from 2.8 to 1106 m, with around 50% of the reaches having lengths below 25 m (Fig. S2). Some reaches consisting of steep waterfalls had lengths below 5 m and a few reaches in almost flat areas were longer than 300 m. The slope in the reaches ranged from 0.01 – 21% and the mean slope was 3% (Fig. S2).

Estimation of stream area

Total stream area was calculated using length and width of the stream reaches. For each location category, daily widths of the streams were calculated using linear regressions developed from the measurements during the propane injections as follows:

L1: $W = 0.002 \times D + 0.92$ (S2)

L2: $W = 0.006 \times D + 0.73$ (S3)

L3: $W = 0.003 \times D + 0.68$ (S4)

L4: assumed to be same as eq. (S3) based on field observations

L5: $W = 0.001 \times D + 0.74$ (S5)

L6: $W = 0.006 \times D + 0.65$ (S6)

L7: $W = 0.0009 \times D + 1.74$ (S7)

where W and D are the width of the stream (m) and stream discharge (L s^{-1}) measured during the propane injections, respectively. Equations (S5) and (S7) were estimated using assumed minimum and maximum width during minimum and maximum discharges, respectively, based on field observations, as no detailed width measurements were made here. Thus, variations in stream area due to variations in discharge were included in our area estimates and the total mean stream area was estimated as 6319 ± 1103 m^2.

Supplementary References

1 Wallin, M. B. *et al.* Spatiotemporal variability of the gas transfer coefficient (KCO_2) in boreal streams: Implications for large scale estimates of CO_2 evasion. *Global Biogeochem. Cycles* **25**, GB3025, doi: 10.1029/2010gb003975 (2011).

2 Jones, J. B. & Mulholland, P. J. Methane input and evasion in a hardwood forest stream: Effects of subsurface flow from shallow and deep pathways. *Limnol. Oceanogr.* **43**, 1243-1250 (1998).

3 Hope, D., Palmer, S. M., Billett, M. F. & Dawson, J. J. C. Carbon dioxide and methane evasion from a temperate peatland stream. *Limnol. Oceanogr.* **46**, 847-857 (2001).

4 Marzolf, E. R., Mulholland, P. J. & Steinman, A. D. Improvements to the Diurnal Upstream–Downstream Dissolved Oxygen Change Technique for Determining Whole-Stream Metabolism in Small Streams. *Can. J. Fish. Aquat. Sci.* **51**, 1591-1599 (1994).

5 Bastviken, D., Sundgren, I., Natchimuthu, S., Reyier, H. & Gålfalk, M. Technical Note: Cost-efficient approaches to measure carbon dioxide (CO_2) fluxes and concentrations in terrestrial and aquatic environments using mini loggers. *Biogeosciences* **12**, 3849-3859 (2015).

6 Weiss, R. F. Carbon dioxide in water and seawater: the solubility of a non-ideal gas. *Mar. Chem.* **2**, 203-215 (1974).

7 Bastviken, D. in *Encyclopedia of Inland Waters* (ed G. E. Likens) 783-805 (Academic Press, 2009).

8 Wiesenburg, D. A. & Guinasso, N. L. Equilibrium solubilities of methane, carbon monoxide, and hydrogen in water and sea water. *J. Chem. Eng. Data* **24**, 356-360 (1979).

9 Wallin, M. B. *et al.* Temporal control on concentration, character, and export of dissolved organic carbon in two hemiboreal headwater streams draining contrasting catchments. *J. Geophys. Res.: Biogeosci.* **120**, 832-846 (2015).

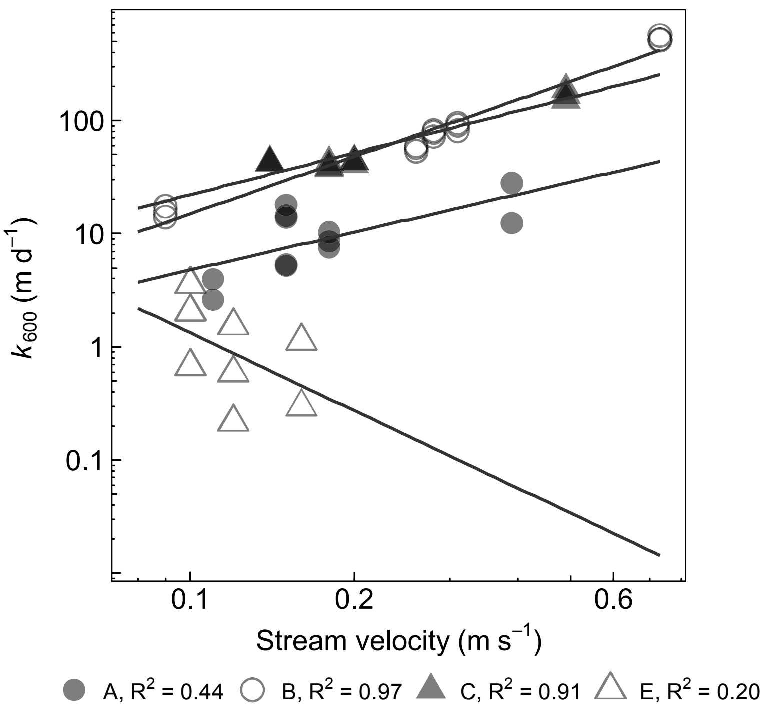

Figure S1. Relationship of k_{600} with stream velocity in the reaches where propane injections were made. The p values for A, B, C and E were 0.018, < 0.001, < 0.001 and 0.261 respectively. D and F were excluded as they were measured only once. Note the $\log_{10}$ scales on both axes.

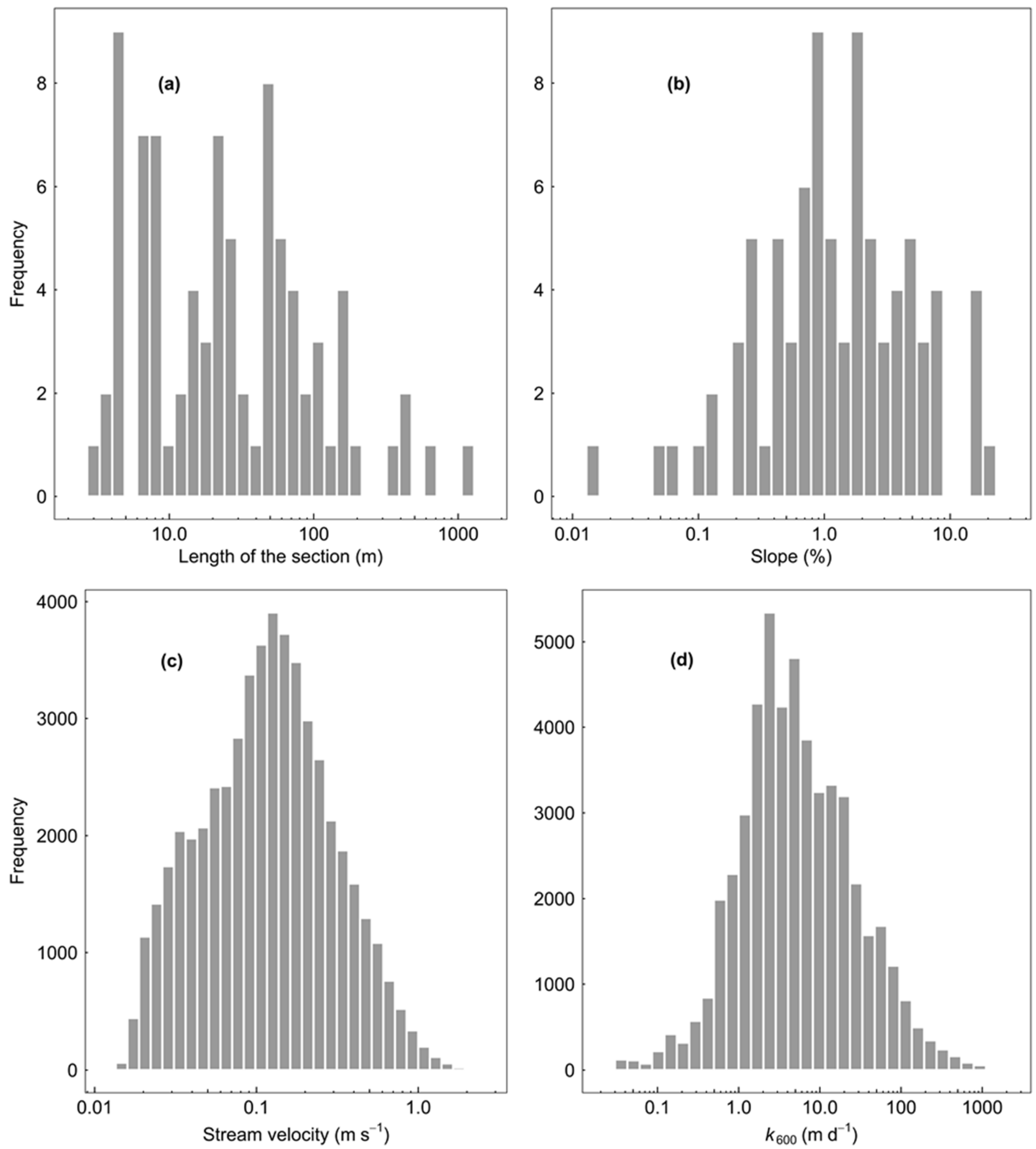

Figure S2. Histograms of length (a) and slope (b) of the 84 reaches of the stream network and the distribution of modelled velocity (c) and k_{600} (d) for all 84 reaches for the two years. Note the $\log_{10}$ x-axis in all panels.

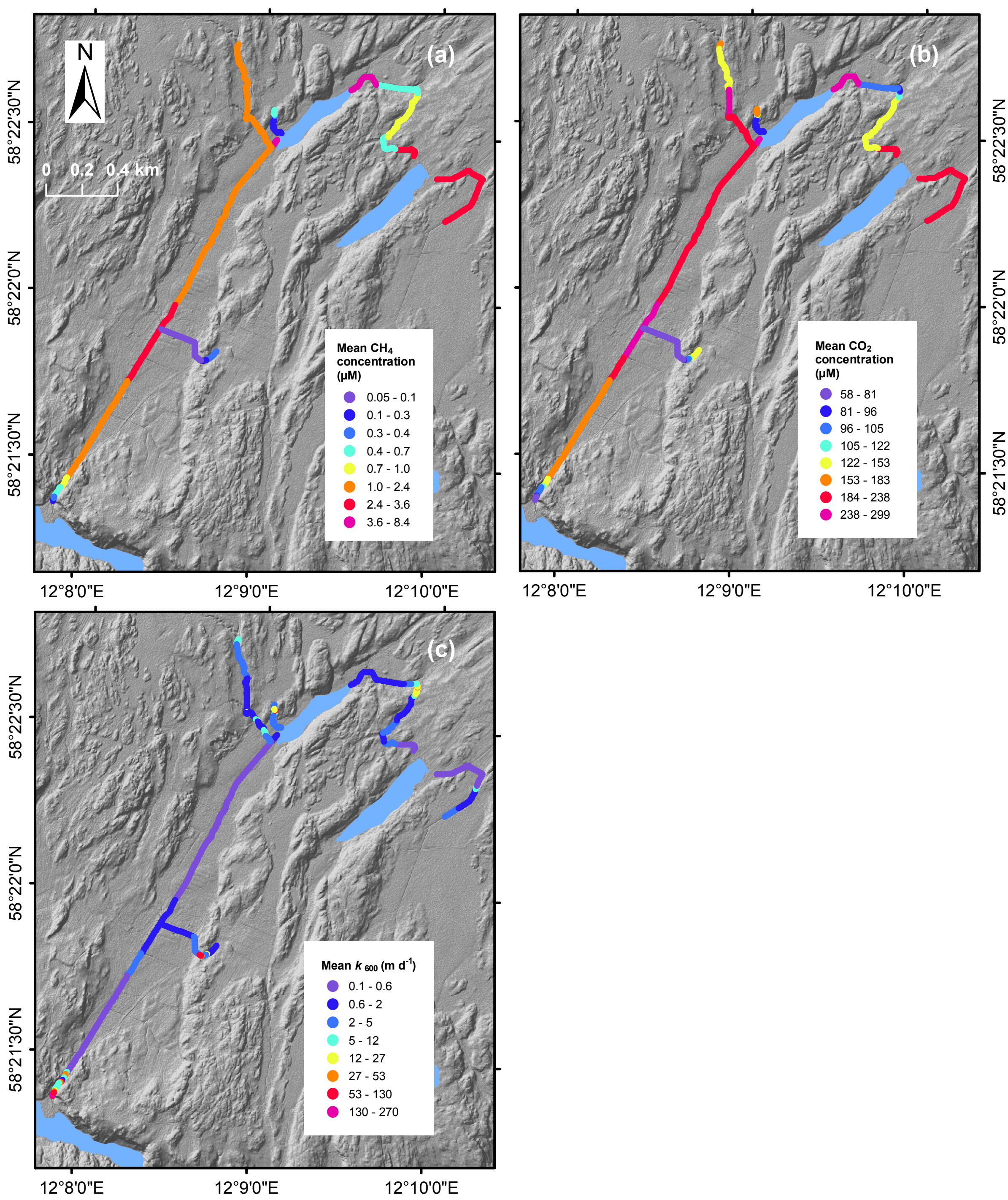

Figure S3. Mean CH_4 concentrations (a), CO_2 concentrations (b) and k_{600} (c) in the studied stream network. The figure was created with ArcMap 10.3.1 available from http://www.esri.com/. The background maps were obtained from Lantmäteriet (National Land Survey of Sweden) and published under the copyright agreement i2012/898 with Linköping University.

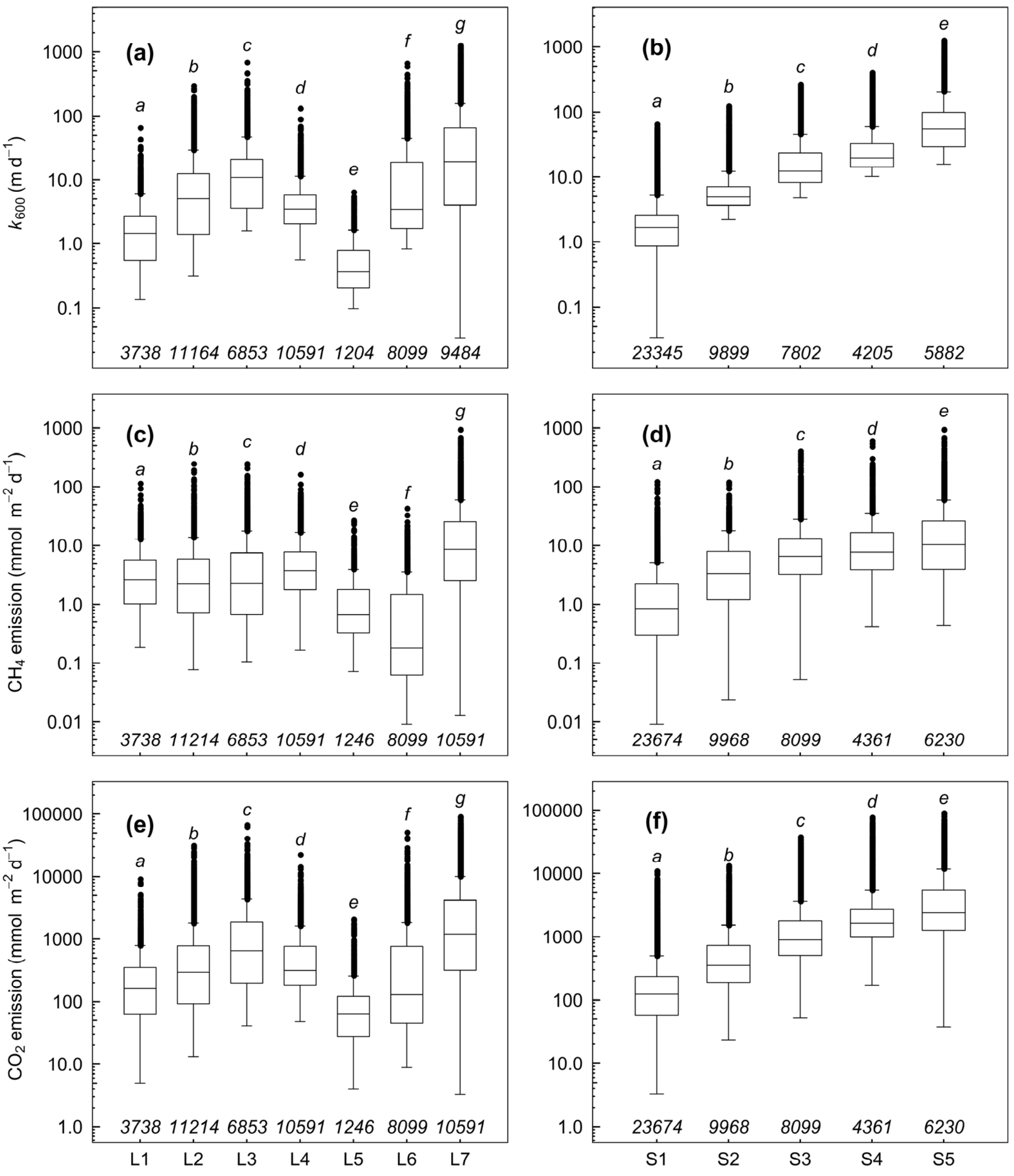

Figure S4. Modelled k_{600} (a, b), CH_4 (c, d) and CO_2 (e, f) emissions in the stream network for the years 2013 and 2014 grouped into location and slope categories. The letters above the boxes represent Tukey's post-hoc test and boxes with different letters had significantly different mean values ($p < 0.05$). The numbers below the boxes are the number of modelled values in each category. Note the log_{10} scale in y-axes.

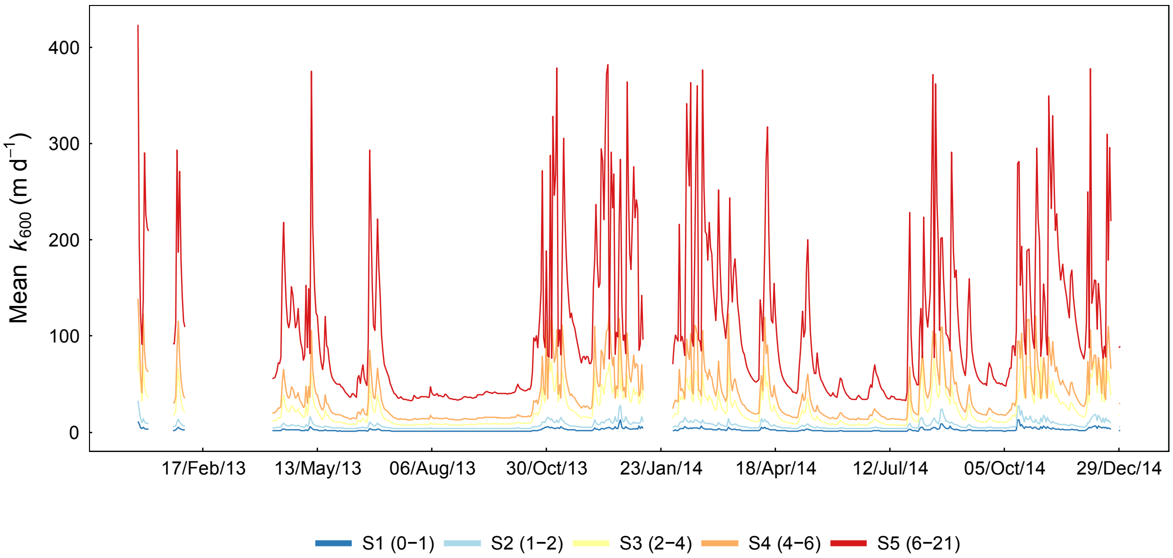

Figure S5. Temporal variations in mean k_{600} for the different slope categories of the stream network, showing the slope dependent spatial variability and the discharge dependent temporal variability in the two years in focus. The numbers in brackets denote the slope interval for each category (%).

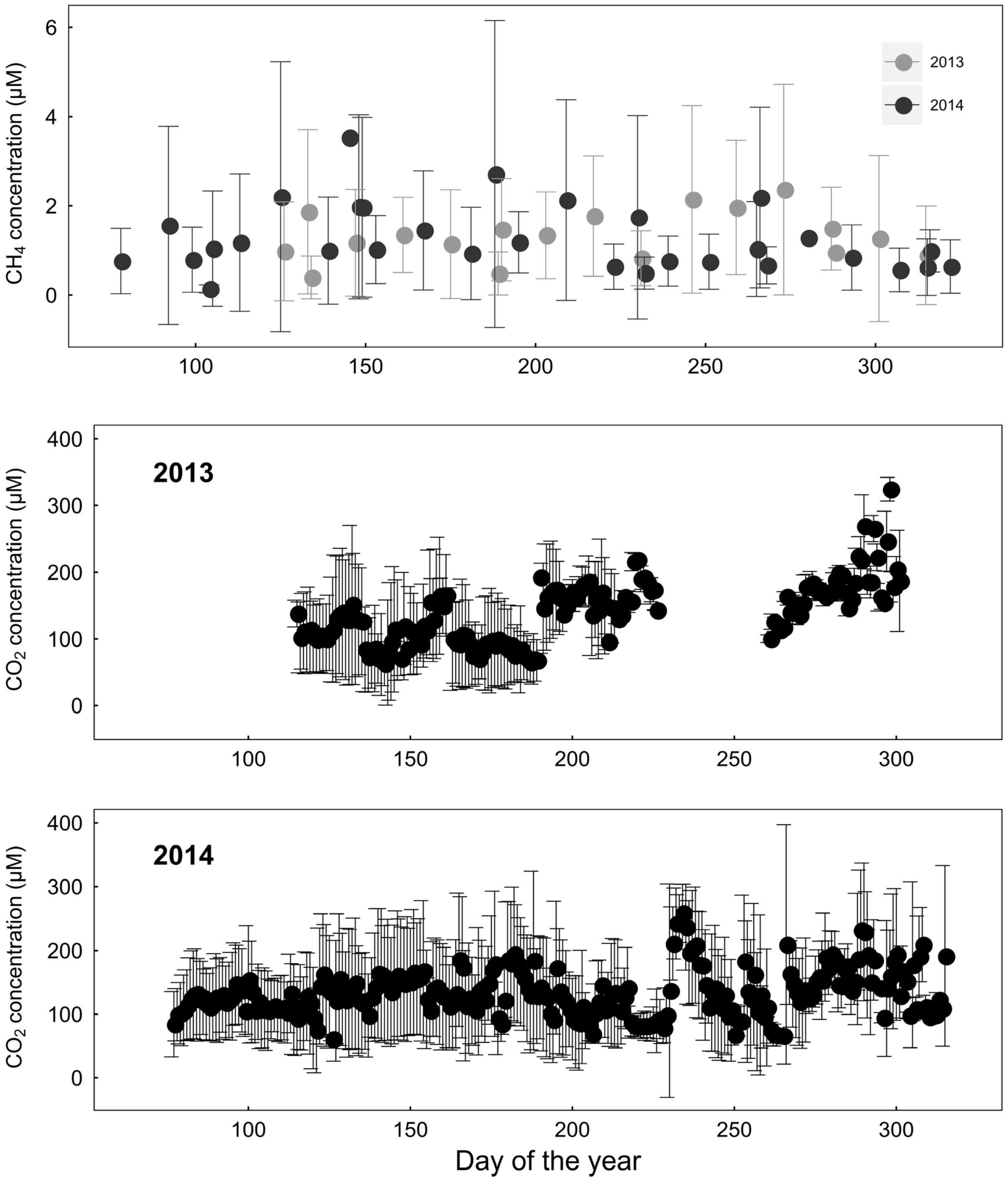

Figure S6. CH_4 and CO_2 concentrations in the streams (mean ± 1SD of all sampling points on any given day) in 2013 and 2014 (top and two bottom panels, respectively). Four high CH_4 concentrations values in the range 9.5 to 46.1 µM were removed from the plot for clarity.

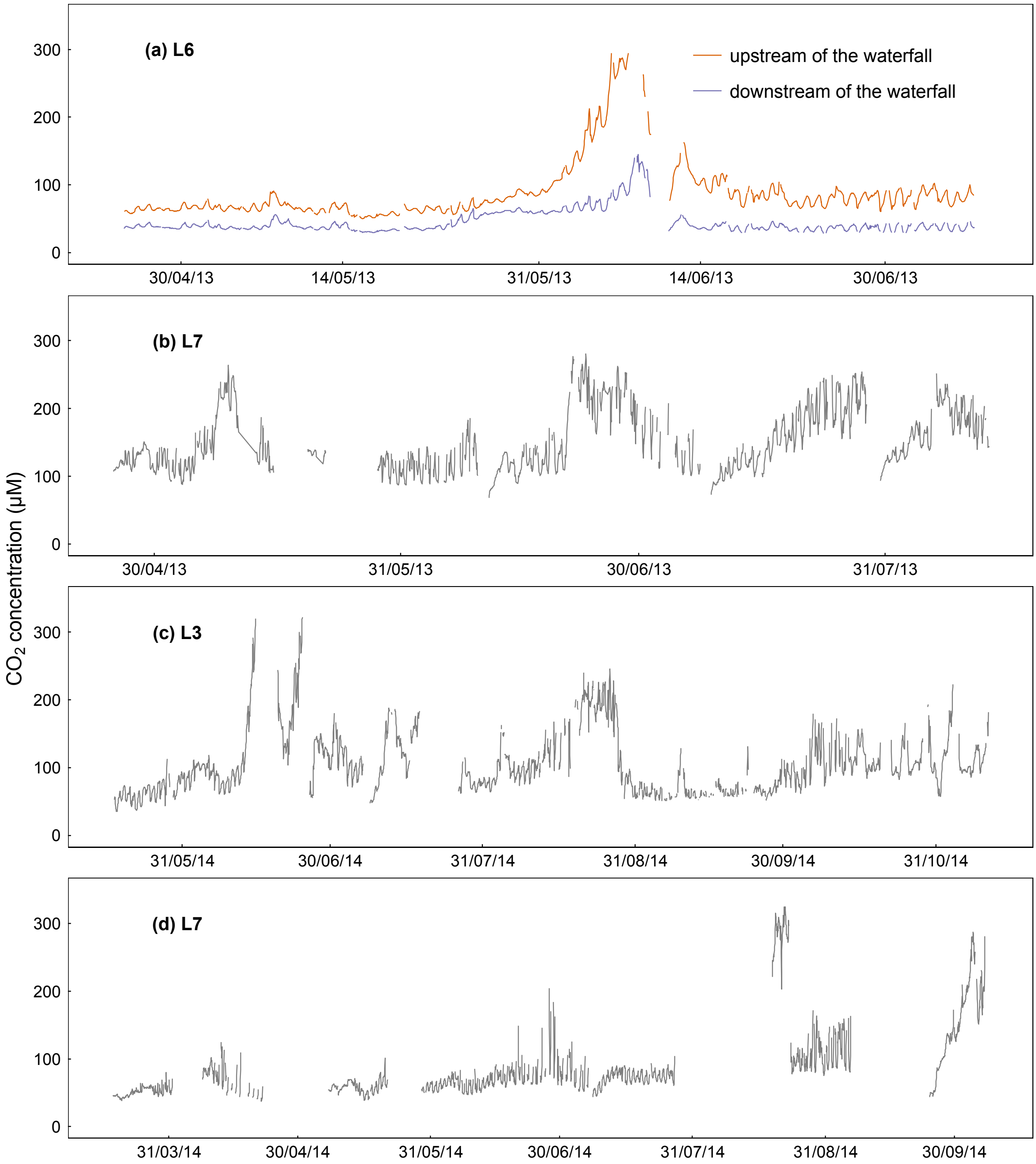

Figure S7. Example data from continuous monitoring of CO_2 in streams from three different locations in the two years. The panel (a) shows CO_2 concentration from above and below a waterfall in L6, showing consistently lower CO_2 in the downstream point and diel variability. Note the different x-axis in each of the panels.

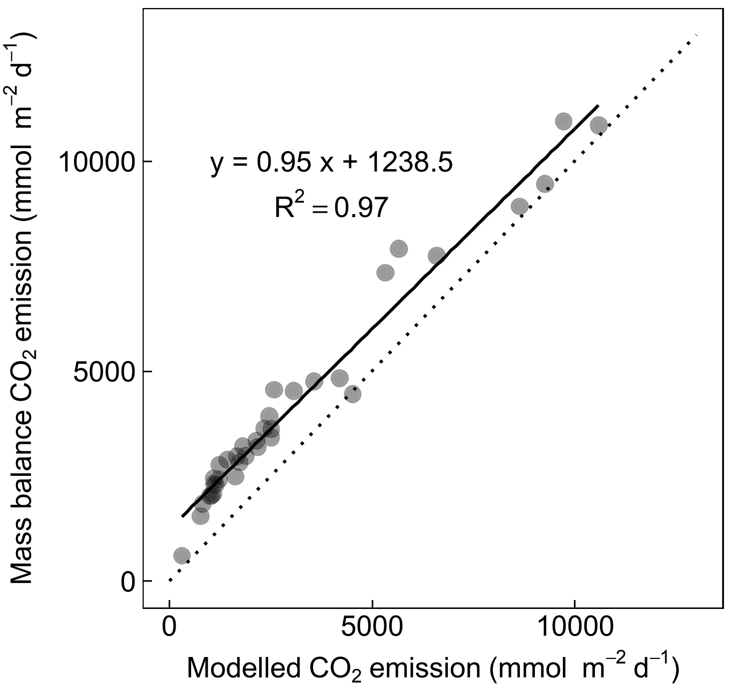

Figure S8. Scatter plot of CO_2 emissions obtained from modelling and the emissions from a mass balance approach (see Supplementary Methods for details) for a turbulent 300 m stream length close to the outlet of lake Skottenesjön.

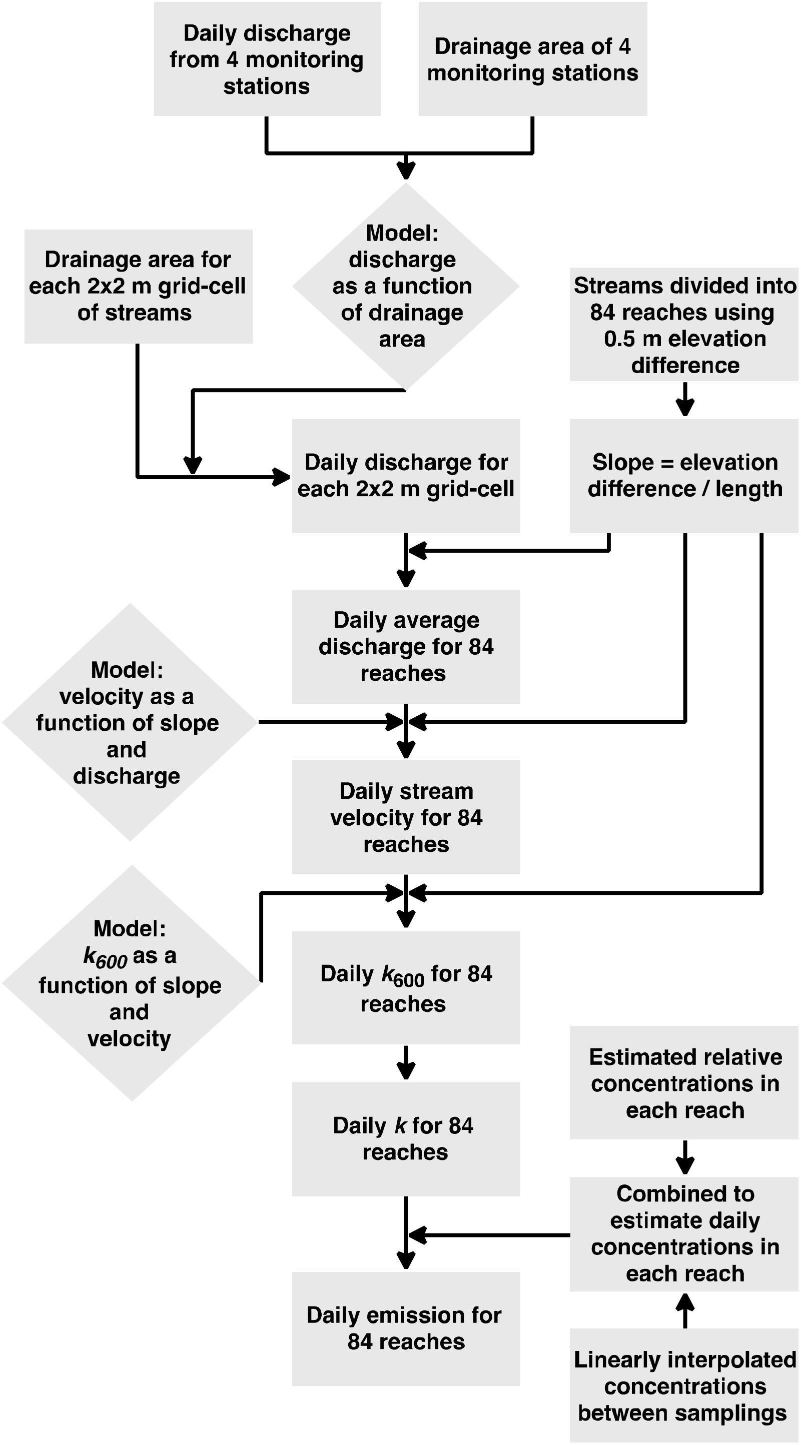

Figure S9. A brief flowchart of the methodology used to estimate emissions for all the 84 reaches of the streams of SRC.

Table S1. Comparison of emissions from chamber measurements and modelled emissions during low flow

Date	Location [a]	Discharge ($L\ s^{-1}$) [b]	Slope (%)	Measured CO_2 emissions ($mmol\ m^{-2}\ d^{-1}$)	Modelled CO_2 emissions ($mmol\ m^{-2}\ d^{-1}$) [c]
18 March 2014	G [d]	66.2	0.1	34.1 [e]	96.0
19 March 2014	H [d]	66.5	0.3	219.7 [e]	258.3
19 March 2014	H	66.5	0.3	252.2 [e]	258.3
19 March 2014	H	66.5	0.3	249.7 [e]	258.3
08 July 2014	I	0.8	0.03	18.9 [f]	11.3
08 July 2014	J	2.0	0.03	12.0 [f]	2.6
08 July 2014	K	3.9	0.03	27.7 [f]	28.8
08 July 2014	H [d]	5.1	0.03	78.9 [f]	54.4
08 July 2014	G [d]	6.4	0.03	24.7 [f]	33.0

[a] for locations see Fig. 1

[b] discharge for each location on specific dates obtained from modelling (see Methods)

[c] see Table 2 for equations

[d] the chambers were drifted for ~ 20 m on 18-19 March; in the same location the chambers were kept static in pools on 8 July

[e] emissions measured by drifting chambers along the stream

[f] emissions from chambers in static pools of water during low flow
